# Improvements in Bacterial Primers to Enhance Selective SSU rRNA Gene Amplification of Plant-associated Bacteria by Applying the LNA Oligonucleotide-PCR Clamping Technique

**DOI:** 10.1264/jsme2.ME18071

**Published:** 2018-09-29

**Authors:** Makoto Ikenaga, Shohei Katsuragi, Yoshihiro Handa, Hiroshi Katsumata, Naoya Chishaki, Tomohiro Kawauchi, Masao Sakai

**Affiliations:** 1 Research Field in Agriculture, Agriculture Fisheries and Veterinary Medicine Area, Kagoshima University 1–21–24, Korimoto, Kagoshima, 890–0065 Japan; 2 Faculty of Agriculture, Kagoshima University 1–21–24, Korimoto, Kagoshima, 890–0065 Japan; 3 Bioengineering Lab. Co., Ltd. Tenko 7th building 5F, 3068 Sakai, Atsugi, 243–0022, Kanagawa Japan; 4 The United Graduate School of Agricultural Sciences, Kagoshima University 1–21–24, Korimoto, Kagoshima, 890–0065 Japan

**Keywords:** PCR clamping, SSU rRNA genes, plant–associated bacteria, LNA oligonucleotide, KU63f and KU1494r

## Abstract

PCR clamping by locked nucleic acid (LNA) oligonucleotides is an effective technique for selectively amplifying the community SSU rRNA genes of plant–associated bacteria. However, the original primer set often shows low amplification efficiency. In order to improve this efficiency, new primers were designed at positions to compete with LNA oligonucleotides. Three new sets displayed higher amplification efficiencies than the original; however, efficiency varied among the primer sets. Two new sets appeared to be available in consideration of bacterial profiles by next-generation sequencing. One new set, KU63f and KU1494r, may be applicable to the selective gene amplification of plant-associated bacteria.

The simultaneous DNA extraction of plant organelle (mitochondria and plastid) SSU rRNA genes is a major limitation associated with amplifying the community SSU rRNA genes of plant–associated bacteria (7, 11, 12, 19, 26). A blocking technique to avoid the concomitant amplification of undesirable sequences (2, 18, 23) and ultra-density gradient centrifugation (9) are useful for obtaining bacterial DNA fractions. Sakai and Ikenaga (21) reported a blocking technique to inhibit the amplification of plant organelle SSU rRNA genes by applying the peptide nucleic acid (PNA)-PCR clamping technique. They subsequently employed locked nucleic acid (LNA) oligonucleotides instead of PNA oligomers to increase the amplification efficiency of plant–associated bacterial SSU rRNA genes (13–15). A PCR clamping technique using LNA oligonucleotides is advantageous because it may selectively amplify almost the full length of bacterial SSU rRNA genes, and amplicons may then be used in a plant microbiome analysis, such as next-generation sequencing (NGS) (27). However, the amplification efficiency of the primer set used in this approach, modified 63f and 1492r, is often low, and this has been attributed to 1) differences in *Tm* values between forward and reverse primers, 2) the double T at the 3′ end of the 1492r primer, and 3) complementary sequences at the 3′ ends of both primers. Furthermore, modified 63f displays lower coverage than the universal primers specific for bacteria (13). To improve amplification efficiency, three new forward primers and one new reverse primer were designed at positions to compete with LNA oligonucleotides specific for plant organelle (mitochondria and plastid) SSU rRNA genes. The LNA oligonucleotides used herein were described in our previous studies (13, 14), and their sequences and properties were shown in supplementary Table S2. The amplification efficiencies of the newly designed primer sets were compared for their respective PCR products prepared from the roots of rice, potato, soybean, and turnip green. NGS was also applied to establish the recommended primer set for microbiome analyses of plant–associated bacteria.

The sequences of bacterial SSU rRNA genes were obtained from representatives of 32 phyla from RDP release 11 (https://rdp.cme.msu.edu) in consideration of phylogenetic diversity. The sequences of type strains were mainly collected. Bacterial genes were aligned together with modified 63f or 1492r. The numbers of bacterial genes used for alignment were 12,392 and 14,290 for the forward and reverse sides, respectively. Based on alignment, three new forward primers, KU63f, KU64f, and KU68f, were designed by improving the modified 63f primer at the position to compete with LNA oligonucleotides on the forward side (Fig. 1A). On the other hand, one new reverse primer, KU1494r, was designed by removing double T at the 3′ end of the 1492r primer on the reverse side (Fig. 1B). The properties of the newly designed primers are listed in Table 1. The original set of modified 63f and 1492r displayed the maximal difference of 10°C in *Tm* values, whereas differences in *Tm* values for the three new sets, KU63f and KU1494r, KU64f and KU1494r, and KU68f and KU1494r, were slightly lower at 5, 7, and 9°C, respectively. Regarding primer coverage, modified 63f has been reported to show 76.3% coverage, and this percentage is lower than that of universal primers specific for bacteria, as calculated using ROSE version 1.1.2 (3). For example, the coverages of the common bacterial primers, 68f, 341f, 517f, 799f, 518r, 805r, 907r, and 1390r, were 74.2, 89.1, 89.9, 81.9, 83.8, 91.1, 84.4, and 89.2%, respectively (supplementary Table S1). However, the newly designed KU63f, KU64f, and KU1494r showed similar coverages of 80.6, 80.0, and 81.7%, respectively. In contrast, KU68f displayed 73.0%, which was lower than that of modified 63f. As shown in Table 2, the modified forward primers 63f, KU63f, and KU64f and reverse primers 1492r and KU1494r have complementary sequences at their 3′ ends, which led to less objective PCR products after the exponential generation of primer dimers. Hot start DNA polymerase is considered to be effective for avoiding this drawback, while KU68f was additionally designed to avoid the formation of primer dimers by shifting the annealing position toward the extension side.

Rice (*Oryza sativa* cv. Koshihikari), potato (*Solanum tuberosum* cv. Nishiyutaka), soybean (*Glycine max* cv. Fukuyutaka), and turnip green (*Brassica rapa* var. perviridis Hamami No. 2) were cultivated in potted soil. Cultivation procedures were the same as those described by Ikenaga *et al.* (13, 14). The roots of rice and soybean were collected at the seedling stage, while those of potato and turnip green were collected at the harvest stage. Respective roots were treated with 0.5% SDS, washed in sterilized distilled water to remove attachments, such as soil particles, and then ground to prepare suspensions containing 0.25 g of fresh roots mL^−1^. DNA extraction was performed from 0.5-mL aliquots of root suspensions using the FastDNA SPIN Kit for Soil (MP Biomedicals, Solon, OH, USA) with a phenol/chloroform treatment. Extracted DNA was stored at −20°C before use.

The SSU rRNA genes of plant–associated bacteria were selectively amplified by applying the PCR clamping technique with the following LNA oligonucleotides for the respective root DNA extracts: Mit63a, Mit1492a, Pla63a, and Pla1492a for rice; Mit63a, Mit1492a, Pla63b, and Pla1492b for soybean; Mit63a, Mit1492a, Pla63c, and Pla1492b for potato, and Mit63b, Mit1492a, Pla63a, and Pla1492b for turnip green (15). The final concentration of each LNA oligonucleotide was 4.0 μM. The sequences of the respective LNA oligonucleotides and their properties were listed in supplementary Table S2. The four combinations of the primer sets used for the respective DNA extracts were as follows: 1) modified 63f and 1492r, 2) KU63f and KU1494r, 3) KU64f and KU1494r, and 4) KU68f and KU1494r. The Premix *ExTaq™* Hot Start Version (Takara, Kusatsu, Japan) was used for PCR, which was performed under the following conditions: 94°C for 3 min as the initial denaturation followed by 35 cycles of 94°C for 30 s, 70°C for 30 s, 54°C for 30 s, and 72°C for 2 min with the final extension step of 72°C for 8 min. Two microliters of PCR products were electrophoresed on a 1.2% agarose gel with a 100-bp marker.

As shown in Fig. 2, PCR products amplified with the original primer set, modified 63f and 1492r, displayed low band intensities for the four types of root samples examined. In contrast, the intensities of the PCR products amplified with the three new primer sets, KU63f and KU1494r, KU64f and KU1494r, and KU68f and KU1494r, were higher. The products of KU68f and KU1494r showed the highest intensity, followed by the products of KU63f and KU1494r and those of KU64f and KU1494r. This order of efficiency appeared to be attributed to the number of complementary bases at the 3′ ends of the forward and reverse primers. As described above, KU68f was designed to avoid the exponential generation of primer dimers. Even though the difference in *Tm* values was 9°C at the maximum between KU68f and KU1494r, this primer set displayed the highest intensities of PCR products. In contrast, KU63f and KU1494r as well as KU64f and KU1494r retained complementary sequences at their 3′ ends (Table 2). However, the five original complementary bases were decreased to three and four complementary bases for KU63f and KU1494r and for KU64f and KU1494r, respectively. Thus, band intensities were inversely proportional to the number of complementary bases. This result suggested that the removal of double T at the 3′ end of 1492r to design KU1494r also contributed to the increase in PCR efficiency.

An NGS analysis for the V3 and V4 regions of bacterial SSU rRNA genes was performed using a paired–end method with MiSeq (Illumina, San Diego, CA, USA). The set of the primers 341f (*Escherichia coli* positions 341–357; 5′-CCT ACGGGNGGCWGCAG-3′) and 805r (*E. coli* positions 805–785; 5′-GACTACHVGGGTATCTAATCC-3′) (16) with adaptor sequences followed by index sequences was used for the respective purified amplicons. The Qiime pipeline (6) was used for a microbiome analysis after removing low quality and chimeric sequences. The sequences that passed preprocessing were clustered in operational taxonomic units (OTUs) at 97% similarity. The procedure described was performed by Bioengineering Lab. (http://www.gikenbio.com/). The abundance rates of the respective bacterial phyla examined with the four primer sets are shown in Fig. 3. The OTUs that accounted for more than 1% of the total sequence number were considered, whereas those that represented less than 1% were classified as “other bacteria”.

As shown in Fig. 3, *Firmicutes* and *Proteobacteria* were the dominant bacterial phyla, with *Actinobacteria* being the next dominant phylum in the four examined plant roots irrespective of the difference in the primer set. *Acidobacteria* were also widespread in turnip green. However, the abundance ratios of the respective bacterial phyla varied depending on the primer sets. The set of KU68f and KU1494r displayed the highest efficiency in PCR amplification; however, *Firmicutes* sequences were more likely to be preferentially amplified with this set, which resulted in a reduction in the detection of other phyla based on the abundance ratio. As shown in Table 3, the coverage of the last four DNA bases at the 3′ end of KU68f, corresponding to positions 65 to 68 in *E. coli*, displayed nearly 80% or consistently higher percentages for *Proteobacteria, Firmicutes*, *Actinobacteria*, TM7, and *Verrucomicrobia*. In contrast, DNA bases showing low coverage in this region were observed for the other phyla; however, all bases of TM6 and two bases of *Planctomycetes* displayed low coverage throughout positions 60 to 68. Bru *et al.* (5) and Wu *et al.* (24) demonstrated that PCR amplification was inhibited if a single mismatch occurred within the last three to four nucleotides of the 3′ end of the primer, even when the annealing temperature was decreased for optimal conditions. Therefore, an amplification bias may have occurred due to a mismatch within the last four bases at the 3′ end. Consequently, *Firmicutes*, which was generally observed as the dominant phylum in the microbiome analysis of plant–associated bacteria as well as *Proteobacteria* (1, 10, 20, 25, 28), was preferentially amplified.

*Firmicutes* and *Proteobacteria* were also observed as the dominant phyla when the primer sets KU63f and KU1494r and KU64f and KU1494r were used (Fig. 3). However, these sets did not show a bias in favor of *Firmicutes*, and the abundance ratios of other phyla were higher than those in the experiment when the primer set KU68f and KU1494r was used. In addition, DNA bases showing low coverage were not detected in the last four bases at the 3′ end, similar to the case of KU68f, except for one base in *Planctomycetes*, suggesting that both primer sets are applicable to investigations of the microbiome of plant–associated bacteria.

The primer set KU63f and KU1494r displayed higher amplification efficiency than original modified 63f and 1492r, and the coverage of KU63f was greater than that of modified 63f. These two primer sets showed more similar abundance ratios not only at the phylum level, but also at more detailed levels such as order, class and genus levels than when KU64f and KU1494r was used (Fig. 3). Data supporting this result was also obtained in a cluster analysis under the Black Box program (http://aoki2.si.gunma-u.ac.jp/bb0/BlackBox0.html) (Fig. 4). This was because KU63f was designed by removing three DNA bases at the 5′ end of modified 63f to increase coverage. Therefore, the remaining sequence was identical between KU63f and modified 63f until the 3′ end. As reported by Bru *et al.* (5) and Wu *et al.* (24), the latter half of the primer sequence at the 3′ end side significantly influenced primer annealing at the objective position during PCR cycles (4, 8, 17, 22). Therefore, these two sets were assumed to yield similar abundance ratios in bacterial profiles. In the present study, we also demonstrated that the coverage of modified 63f was lower than that of other universal bacterial primers. However, since lower coverage was caused by three DNA bases at the 5′ end of modified 63f, this primer was not expected to give a stronger bias in PCR amplification than that estimated from the calculated coverage. The plant microbiomes prepared with the KU63f and KU1494r and modified 63f and 1492r primers sets may be compared, even with this difference in PCR efficiency.

In summary, three new forward primers and one new reverse primer were designed at positions to compete with LNA oligonucleotides by improving the original primer set of modified 63f and 1492r in order to selectively PCR-amplify the SSU rRNA genes of plant associated–bacteria. All new primer sets displayed higher amplification efficiencies than that of the original set. However, these efficiencies varied depending on the primer sets; KU68f and KU1494r showed the highest intensity of PCR products, followed by the products of KU63f and KU1494r and those of KU64f and KU1494r. The use of the primer set KU63f and KU1494r 1) did not show a bias in favor of *Firmicutes*, as observed with KU68f and KU1494r, and 2) displayed higher amplification efficacy than KU64f and KU1494r. Consequently, KU63f and KU1494r may be applicable to the selective gene amplification of plant–associated bacteria.

The nucleotide sequences obtained in the present study were registered to Genebank under accession number PRJDB6970.

## Supplementary Material



## Figures and Tables

**Fig. 1:**
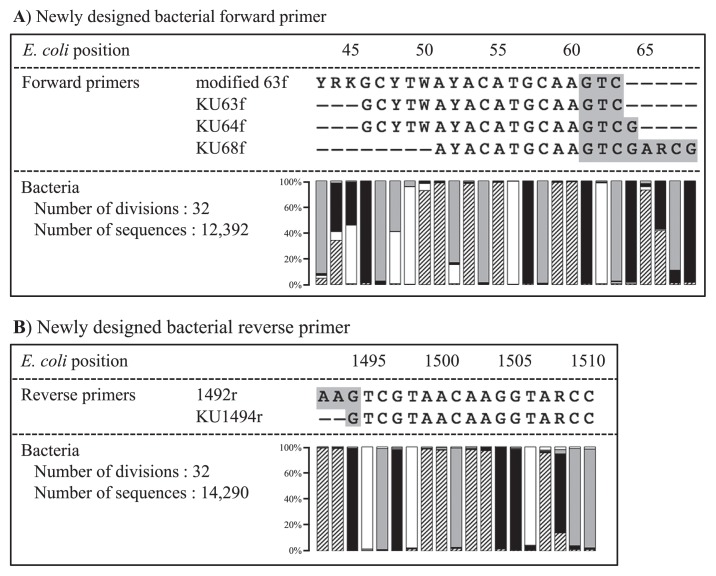
Sequence alignment data of original primer and bacterial SSU rRNA genes to design new bacterial primers at a position to compete with LNA oligonucleotides Highlighted DNA bases in grey indicate the region competing with LNA oligonucleotides. Symbols ▨, □, ■, and 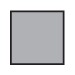 indicated A, T, G, and C, respectively.

**Fig. 2:**
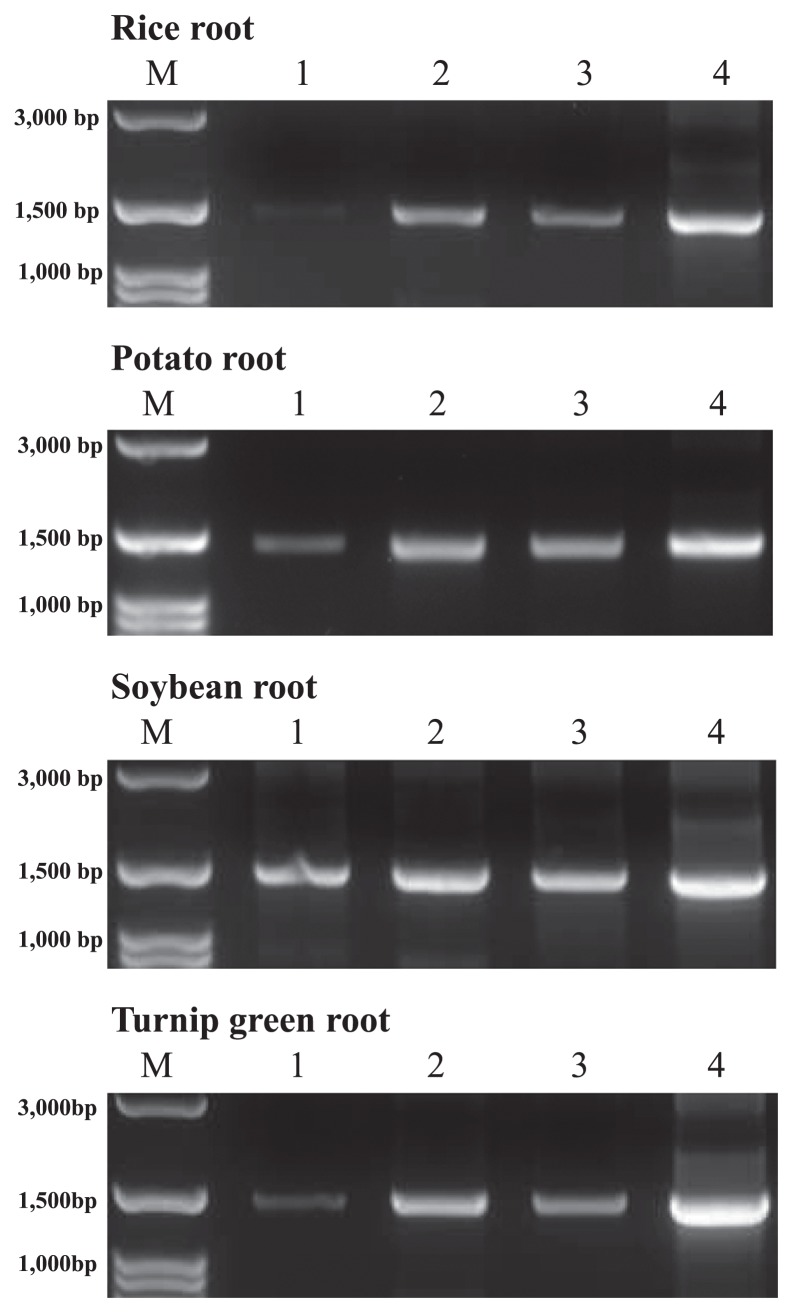
PCR products of respective root–associated bacterial SSU rRNA genes by applying the PCR clamping technique by LNA oligonucleotides Lanes 1, 2, 3, and 4 indicate the products amplified with modified 63f and 1492r, KU63f and KU1494r, KU64f and KU1494r, and KU68f and KU1494r, respectively.

**Fig. 3:**
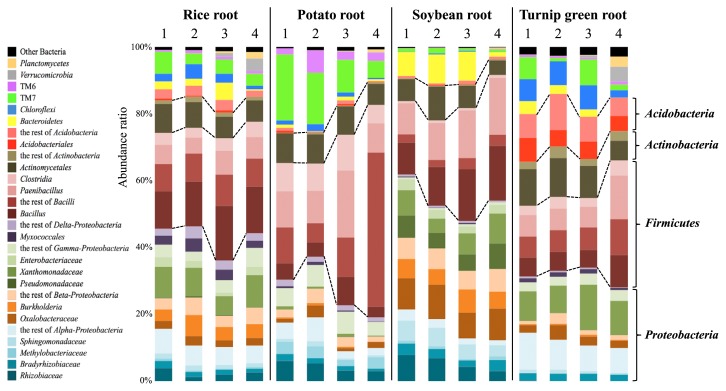
Abundance ratio (%) of plant root–associated bacteria. Phyla accounting for less than 1% were compiled as “other bacteria”. Lanes 1, 2, 3, and 4 indicate the microbiomes prepared by modified 63f and 1492r, KU63f and KU1494r, KU64f and KU1494r, and KU68f and KU1494r, respectively.

**Fig. 4:**
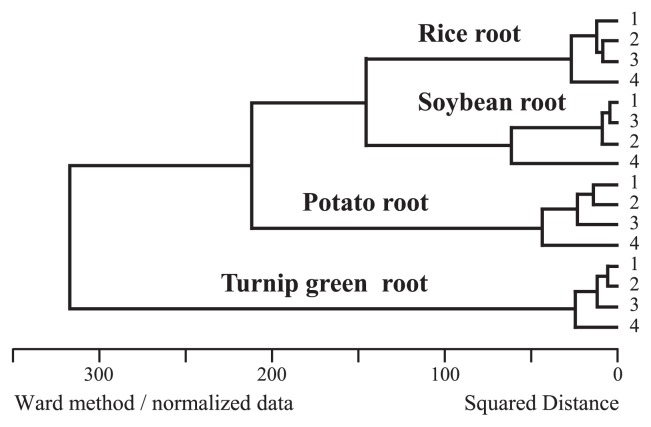
Cluster analysis of respective root–associated bacteria The figure was created based on the NGS profiled in Fig. 3. Lanes 1, 2, 3, and 4 indicate the microbiomes prepared by modified 63f and 1492r, KU63f and KU1494r, KU64f and KU1494r, and KU68f and KU1494r, respectively.

**Table 1:** Properties of newly designed bacterial primers, which were designed by improving the original primer set of modified 63f and 1492r

Primer	5′-Sequence-3′	*Tm* value	Coverage	Size	Position in *E. coli*	References
Forward Primer						
modified 63f	YRKGCYTWAYACATGCAAGTC	58–67°C	76.3%	21mer	43–63	Ikenaga and Sakai (13)
KU63f	GCYTWAYACATGCAAGTC	54–60°C	80.6%	18mer	46–63	This study
KU64f	GCYTWAYACATGCAAGTCG	57–62°C	80.0%	19mer	46–64	This study
KU68f	AYACATGCAAGTCGARCG	58–64°C	73.0%	18mer	51–68	This study

Reverse Primer						
1492r	GGYTACCTTGTTACGACTT	57–60°C	81.2%	19mer	1510–1492	Ikenaga and Sakai (13)
KU1494r	GGYTACCTTGTTACGAC	55–58°C	81.7%	17mer	1510–1494	This study

Y=C or T; R=A or G; K=T or G; W=A or T

**Table 2:** Positions of complementary sequences at the 3′ end between forward and reverse primers

Primer	Sequence																																				
modified 63f	5′	Y	R	K	G	C	Y	T	W	A	Y	A	C	A	T	G	C	A	A	G	T	C	–	3′													
KU63f				5′	G	C	Y	T	W	A	Y	A	C	A	T	G	C	A	A	G	T	C	–	3′													
KU64f				5′	G	C	Y	T	W	A	Y	A	C	A	T	G	C	A	A	G	T	C	G	3′													
KU68f									5′	A	Y	A	C	A	T	G	C	A	A	G	T	C	G	A	R	C	G	3′									

1492r																	3′	T	T	C	A	G	C	A	T	T	G	T	T	C	C	A	T	Y	G	G	5′
KU1494r																	3′	–	–	C	A	G	C	A	T	T	G	T	T	C	C	A	T	Y	G	G	5′

“ 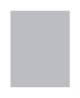
” indicates the positions showing complimentary sequences.

**Table 3:** Coverage (%) of DNA bases at the 3′ end position of forward primers in respective bacterial phyla, which constituted more than 1% in the NSG analysis

*E. coli* position	60	61	62	63	64	65	66	67	68
DNA base	A	G	T	C	G	A	R	C	G
*Proteobacteria*	92.8	93.9	94.4	94.3	94.6	87.1	94.1	94.2	94.7
*Firmicutes*	85.8	87.4	87.7	82.5	90.1	86.6	90.4	86.3	84.7
*Actinobacteria*	94.7	95.3	95.8	95.6	96.1	95.1	96.4	96.7	97.1
*Acidobacteria*	81.3	84.8	84.8	86.1	86.1	59.1	88.7	86.1	87.0
*Bacteroidetes*	89.0	92.9	83.3	93.1	93.6	85.6	93.9	38.9	95.4
*Chloroflexi*	88.5	92.3	91.3	91.3	92.8	73.6	91.8	92.3	91.8
TM7	86.9	86.4	92.8	87.7	90.3	84.7	90.7	85.2	89.8
TM6	58.3	60.4	58.3	58.3	59.4	59.4	60.4	58.3	59.4
*Verrucomicrobia*	85.9	87.6	88.0	86.7	88.4	79.5	87.6	87.6	88.0
*Planctomycetes*	53.3	93.1	93.7	93.3	96.2	37.4	96.0	85.1	83.8
