# Supplementary material for: Improvements in Bacterial Primers to Enhance Selective SSU rRNA Gene Amplification of Plant-associated Bacteria by Applying the LNA Oligonucleotide-PCR Clamping Technique
Source: Microbes Environ. 2018 Sep 29;33(3):340–4. doi: 10.1264/jsme2.ME18071 (PMC6167120; doi:10.1264/jsme2.ME18071)
Supplement: Supplementary file 1 [file 33_340_s1.pdf]

Supplementary Table S1 Sequences and coverganes of common bacterial primers

Primer	Position of digenerate base	5'–Sequence–3'	Coverage	Position in *E.coli*	References
68f	2N4N17R18R	TNANACATGCAAGTCGRRCG	74.2%	49–68	Thijs et al. 2017. Front. Microbiol. 28:494.
341f	9N13W	CCTACGGGNGGCWGCAG	89.1%	341–357	Thijs et al. 2017. Front. Microbiol. 28:494.
517f		GCCAGCAGCCGCGGTAA	89.9%	517–533	Wang and Qian 2009. PLoS One 29:e7401.
799f	4M18K	AACMGGATTAGATACCCKG	81.9%	781–799	Ghyselinck et al. 2013. PLoS One 8:e71360.
518r		ATTACCGCGGCTGCTGG	83.8%	534–518	Ghyselinck et al. 2013. PLoS One 8:e71360.
805r	7H8V	GACTACHVGGGTATCTAATCC	91.1%	805–785	Thijs et al. 2017. Front. Microbiol. 28:494.
907r	11M	CCGTCAATTCMTTTGAGTTT	84.4%	926–907	Mao et al. 2012. BMC Microbiol. 12:66.
1390r	14R	ACGGGCGGTGTGTRCAA	89.2%	1406–1390	Mao et al. 2012. BMC Microbiol. 12:66.

Coverage (%) was calculated with the ROSE version 1.1.2.

Supplementary Table S2 Sequences of respective LNA oligonucleotides and their properties

Plant organelle	LNA oligonucleotide	5'–Sequence–3'	Size	*Tm* value
Mitochondria	LNA-Mit63a	gtcgaAcgTtgtTTtCgG-p	18 bases	70 °C
	LNA-Mit63b	gtcgaAcgTtGttCccTg-p	18 bases	70 °C
	LNA-Mit1492a	cttcAccccaGtcgAAgA-p	18 bases	70 °C
Plastid	LNA-Pla63a	gtcgAacggGaagTggT-p	17 bases	70 °C
	LNA-Pla63b	tcgGacgggaaGTggT-p	16 bases	70 °C
	LNA-Pla63c	tcgGacgggaaACAcg-p	16 bases	71 °C
	LNA-Pla1492a	cttcacTccaGtcgcAagC-p	19 bases	70 °C
	LNA-Pla1492b	cttcacTccaGtcaCTagC-p	19 bases	71 °C

Small letter and capital letter in the sequence indicated the DNA base and LNA base, respectively. The 3' end of all LNA oligonucleotides were phosphorylated to avoid the extension during PCR. It was indicated as "p" in the sequences.
